# Advance Market Commitments and Their Role in Public Innovation

**DOI:** 10.1017/jme.2025.10153

**Published:** 2025

**Authors:** Sarosh Nagar, Anil Cacodcar, Aaron S. Kesselheim

**Affiliations:** 1 Program On Regulation, Therapeutics, And Law (PORTAL), Division of Pharmacoepidemiology and Pharmacoeconomics, Department of Medicine, Brigham and Women’s Hospital, Boston, MA; 2 University College London, London, UK; 3 Harvard Medical School, Boston, MA; 4 Harvard College, Cambridge, MA

**Keywords:** Science funding, Innovation policy, Metascience, Advance market commitment, Biomedical research

## Abstract

Advance market commitments (AMCs) are gaining increasing attention as an alternative science funding mechanism to promote innovation in medicine. In this paper, we first review the theory underlying AMCs, before analyzing two case studies of prior AMCs: the Gavi, the Vaccine Alliance pneumococcal conjugate vaccine AMC launched in 2007 and the use of AMC-like mechanisms in Operation Warp Speed in the US. We identify the empirical successes and limitations of AMCs in promoting research and development into new therapeutics and vaccine candidates, highlighting both the strong promise of AMCs and the need to complement them with other science funding mechanisms to promote innovation. We conclude with a series of recommendations to inform science policymakers.

While investments in some therapeutic areas can be extremely lucrative for for-profit manufacturers, others may not offer substantial or predictable revenues.^1^ Whether because there are extremely small numbers of patients with the target disease or the target disease is prevalent among populations that cannot pay, in these cases, the development of novel treatments is more challenging. When such a situation involves an unmet medical need, public health is particularly affected by insufficient private investment.

Antibiotics exemplify some of these challenges. Antibiotics are cheap to manufacture and many generic antibiotics that are not protected by patents sell at low prices. Current antibiotics in widespread clinical use are often very effective, and at the same time, doctors are discouraged from overprescribing them to evade resistance.^2^ This combination of factors makes attracting private investment in new antibiotic development difficult, since a new antibiotic would likely be reserved for second- or third-line treatment.^3^ While private manufacturers may not see profit-maximizing potential in new antibiotics, antibiotic-resistant diseases remain a significant public health concern.^4^ Antibiotics represent an example of an “externality,” in which a given product or innovation has some external societal benefit that private-market actors do not internalize, exacerbating the underinvestment problem.^5^ Such externalities are not limited to antibiotics, either — for example, firms also often lack the market incentive to invest in tropical diseases that impact millions in low-income settings, where individuals and health care systems lack the necessary purchasing power for expensive vaccines and treatments.^6^

How can governments close the gap between a firm’s private benefit and society’s public benefit? One option is to have the public sector support drug development. The National Institutes of Health (NIH) have already played critical roles in the development of gene therapies, many transformative drugs, and the COVID-19 vaccine.^7^ Another option is to provide extra private sector incentives in the form of targeted subsidies.^8^ Distinct (though sharing similarities) with this latter option are advance market commitments (AMCs). AMCs occur when the government agrees to preorder a new product in advance of its development, usually in large quantities.^9^ This government purchase sends a clear signal to firms that there is a certain demand for the product, driving investment that may eventually lead to the development of a new drug or innovation.^10^

In recent years, public interest in Kremer’s initial proposal of AMCs as mechanisms of science funding has risen. The University of Chicago recently launched a Market Shaping Accelerator under Kremer’s directive, with advisory board members from academia and the public and private sectors.^11^ Private companies like Stripe and McKinsey launched an environmental technology AMC centered around the development of new carbon capture and removal technologies.^12^ With so much interest in AMCs, it is incumbent to understand the most recent empirical evidence related to this model of science funding. Thus, in this paper, we review two notable cases of AMCs with different designs — the 2009 vaccine AMC from Gavi, the Vaccine Alliance and the US government’s use of AMC-like mechanisms in Operation Warp Speed in 2020 — for evidence of how AMCs have functioned in practice. We conclude by outlining a way to use AMCs to promote the development of socially vital innovation in health care.

## AMCs: Theory and Implementation

Originally proposed by Nobel Prize winner Michael Kremer and Rachel Glennerster in 2004, an AMC is a contract between a donor — such as government, international bodies, or private firm — and a firm capable of bringing a new therapeutic to market.^13^ The contract is a commitment by the donor to purchase a bulk quantity of the future pharmaceutical product once it is brought to market, which creates a strong financial incentive for the firm to invest in the development of that product.^14^ In this way, AMCs can be used by governments and international bodies to incentivize the development of pharmaceutical products, especially those with high social returns but low private returns, like the aforementioned case of antibiotics.^15^

A potential problem with an AMC is that if one or two firms end up developing the new drug or innovation desired in the AMC, those firms could price the product at a high level using their market power.^16^ That would benefit the firms, but if the goal is to produce a drug for a disease for socioeconomically disadvantaged populations worldwide, then high prices might inhibit access to the drug. Therefore, in describing the AMC, Kremer and Glennerster introduced the idea of a “price cap.”^17^ In exchange for the AMC, the donors making the bulk purchase would require firms producing the product to set the price of the innovation close to the marginal cost of making a new dose of the product.^18^ This price cap ensures that if a couple of firms manage to develop an invention, they do not use their market power to excessively price the innovation.^19^

There have been multiple high-profile examples of AMCs being used to spark socially desirable therapeutic innovation in the past two decades, and of these, we selected two as case studies for further review. These two case studies were the AMC for pneumococcal vaccines undertaken by the Gates Foundation and the governments of five nations in 2007 and the AMC mechanisms used by the US during the COVID-19 pandemic as part of its Operation Warp Speed effort to develop new vaccines against COVID-19.^20^

### Pneumococcal Vaccines

One early notable use case of AMCs came in 2007 when five countries — the UK, Russia, Italy, Canada, and Norway — joined forces with the Bill and Melinda Gates Foundation on an AMC for pneumococcal conjugate vaccines.^21^ Pneumococcal disease is a deadly respiratory illness caused by the bacterium *Streptococcus pneumoniae,* which the WHO estimated in 2003 killed over one million children annually, especially children living in low-income areas worldwide.^22^ At the time of the AMC, there was one viable vaccine against pneumococcal disease, made by Wyeth, but the vaccine had a high price and had to be made via a complex manufacturing procedure suited to high-income countries, which prevented its use in lower-income countries.^23^ It is worth noting that GSK and Pfizer entered the market with their own vaccines just prior to the launch of the AMC, while the Serum Institute of India did once the AMC had launched.^24^

The first objective of this AMC was to encourage the research and development of new pneumococcal conjugate vaccines that could easily be manufactured.^25^ The second and third objectives were to increase the supply of pneumococcal conjugate vaccines and the rate of vaccination coverage worldwide, while the fourth objective was to test the efficacy of the AMC mechanism itself.^26^ To achieve this mission, the partner nations and the Gates Foundation offered US$1.5 billion to help fund the development of new pneumococcal conjugate vaccines and expand vaccine uptake in 73 AMC-eligible countries worldwide.^27^ This money was deployed partly by the 5 partner nations agreeing to pay manufacturers for each pneumococcal conjugate vaccine dose they produced, “topping up” the price of the dose in the form of a per-dose subsidy.^28^ This subsidy was supposed to incentivize firms to develop new pneumococcal conjugate vaccines and produce them for the global market. As part of this structure, manufacturers agreed to a price cap to prevent monopoly pricing, with the price of the new vaccines in the AMC being US$3.50 per dose.^29^

The AMC did not appear to substantially increase research and development into new pneumococcal conjugate vaccines, as during the years of the AMC, only one new pneumococcal conjugate vaccine was made from the Serum Institute of India.^30^ There are numerous potential reasons why such innovation never arose; for example, the level of investment by the donors could have been insufficient or pneumococcal conjugate vaccine research might have been technically infeasible for other reasons. However, the purchase commitment helped spur GlaxoSmithKline, Pfizer, and the Serum Institute of India to increase the production of existing pneumococcal conjugate vaccine.^31^ This increase in vaccine supply meant that, by 2020, enough pneumococcal conjugate vaccine doses annually were being distributed worldwide to vaccinate 50 million children each year.^32^ Empirical analysis also showed that in Gavi, the Vaccine Alliance countries eligible to be part of the AMC, rates of pneumococcal conjugate vaccine coverage caught up and later exceeded the coverage rate in the rest of the world.^33^

### Operation Warp Speed

In May 2020, as the COVID-19 pandemic was unfolding, the US government launched Operation Warp Speed, a public-private partnership, to accelerate the development of a new, urgently needed vaccine for COVID-19.^34^ Operation Warp Speed involved many different initiatives, including supporting pharmaceutical companies’ research into vaccine candidates and having the Department of Defense aid in vaccine deployment.^35^

One of its most central features, however, was its use of AMC-like mechanisms. In Operation Warp Speed, the federal government signed AMC-like contracts with different pharmaceutical companies, agreeing to purchase bulk quantities of vaccine doses from each company before the vaccine was actually FDA-approved.^36^ For example, the US government agreed to pay Pfizer US$2 billion for 100 million doses once Pfizer had made a working vaccine.^37^ Rather than opting for a price cap mechanism in which all participating firms faced the same upper limit, the federal government placed multiple purchase orders, with the price changing each time, suggesting that the government prioritized the development of new vaccines over fears of monopoly power for COVID-19 vaccine manufacturers.^38^ Operation Warp Speed’s purchase orders for COVID-19 vaccines totaled US$29.2 billion dollars.^39^

Operation Warp Speed thus provided a strong demand signal that helped incentivize firms to invest in researching COVID-19 vaccines.^40^ While demand for COVID-19 vaccines was high during the pandemic, much of this demand was disaggregated across individual firms and producers, and it was unclear how the government would buy COVID-19 vaccines for the general US population. The AMC-like commitments of Operation Warp Speed aggregated societal demand in a single pre-market contract signed by the government, establishing a clear mechanism for the government to purchase COVID-19 vaccines.^41^ Thanks to this investment, the first Operation Warp Speed-funded vaccine beat analyst expectations for market arrival and received emergency use authorization in December 2020.^42^ The speed of this vaccine research was integral to saving millions of lives. In addition, Operation Warp Speed’s efforts also helped greatly accelerate vaccine distribution through efforts like the concurrent manufacturing of vaccines with clinical trials, concurrent shipping of ancillary kits for vaccination, and more.^43^ The AMC-like mechanism may have played a valuable role here, as its purchase orders provided companies with certain financial results even if vaccine candidates failed, enabling them to scale up manufacturing for fast distribution.^44^

However, there are some reasons to be cautious about attributing this fast research and development solely to the AMC-like mechanisms of Operation Warp Speed. First, due to the ongoing COVID-19 pandemic, there was already extremely high demand for potential COVID-19 vaccines, which means that the amount of extra demand the Operation Warp Speed AMCs signaled was likely limited. Second, other federal investments in basic scientific research also played a critical role — as the upwards of US$200 million spent by the NIH on COVID-19 vaccine-related research highlights, such basic research is critical as a foundation upon which the AMC can act to motivate the final development of the vaccine.^45^ Indeed, for the vaccine, several of the key innovations, like the lipid nanoparticle delivery mechanism, had been supported by decades of extensive publicly funded research which made it possible to commercialize in a quick period.^46^ In turn, it is likely the case that absent such public funding of scientific research, Operation Warp Speed’s efforts may have been slower. Third, on the distribution side, Operation Warp Speed’s AMC-like mechanisms may have helped provide financial certainty for firms to expand manufacturing, but it is worth noting that these AMC-like mechanisms were only one of many potent tools the government used to enhance distribution.^47^

## Policy Recommendations

The evidence from our two case studies provides some insight into the usefulness of AMCs. The Gavi AMC for pneumococcal vaccines empirically had a limited effect on vaccine research but accelerated vaccine deployment worldwide.^48^ The AMC-like mechanisms of Operation Warp Speed likely did help accelerate both vaccine research and deployment in the US.^49^

These cases studies show that AMCs can have important benefits, albeit with some caveats. First, evidence from both our case studies highlights that AMCs can accelerate vaccine deployment in several ways.^50^ At the planning level, evidence suggests that advance market commitments enabled policymakers to have certainty about how the government would purchase the vaccines in advance, enabling better policy planning for therapeutic and vaccine rollout, as seen with Operation Warp Speed.^51^ At the incentives level, the top-up per-dose subsidy model proscribed in the pneumococcal vaccine case appeared to create a strong incentive for firms to more rapidly manufacture and deploy pneumococcal vaccines in AMC-covered countries, as evidenced by the fact that pneumococcal vaccine coverage rates rapidly converged with the global average in AMC nations.^52^

Second, at the level of vaccine research, there remains more uncertainty about the impact of AMCs. The pneumococcal vaccine case highlights that only one new pneumococcal vaccine from Serum Institute of India was introduced during the AMC period.^53^ This disappointing result would initially appear to suggest that AMCs are less effective for the purpose of incentivizing research than previously thought. However, there are other reasons such a takeaway may not be correct. For example, the pneumococcal vaccine AMC may not have been of sufficient size to induce a strong demand signal and truly speed up research. Such an interpretation is supported by the evidence that the much larger Operation Warp Speed AMC-like mechanisms did appear to substantially accelerate COVID-19 vaccine research, though existing demand for COVID-19 vaccines and other factors limit the strength of this evidence.^54^ Together, this result suggests that AMCs can have an impact on vaccine research, though further evidence from upcoming AMCs may help provide a more definitive conclusion.

Third, as the Operation Warp Speed case highlights, to truly ensure AMCs accelerate therapeutic or vaccine research and development, complementary supporting science funding mechanisms, such as basic research grants, are likely necessary in combination with an AMC.^55^ The Operation Warp Speed example highlighted that these complementary mechanisms, such as pre-existing basic research, played a significant role in laying the foundation for the late-stage COVID-19 vaccine research which the Operation Warp Speed AMC-like mechanisms helped catalyze.^56^ In this sense, AMCs should be seen as only part of the solution to accelerate research and coverage rates for cases of positive externalities in medicine.

## Conclusions

The empirical successes of advance market commitments suggests that AMCs can be a powerful tool that governments, international entities, and firms can use to support the development and distribution of therapeutics and vaccines for diseases that private market firms may neglect on their own. However, empirical evidence also suggests that, to be successful, AMCs must not only be of a significant enough size, but be supplemented by existing basic research efforts in order to successfully spur new research and innovation. Together, the results suggest that policymakers should view AMCs as a positive but nuanced tool that must be part of a larger strategy to foster innovation in socially valuable domains. Policymakers should consider exploring potential AMCs for other diseases and illnesses, but be careful to combine these tools with other effective interventions to promote innovation.
